# Predictive Extrinsic Factors in Multiple Victim Shootings

**DOI:** 10.1007/s10935-020-00602-3

**Published:** 2020-07-15

**Authors:** Daniel Ruderman, Ellen G. Cohn

**Affiliations:** 1 The Ellison Institute for Transformative Medicine, University of Southern California, Los Angeles, CA 90089 USA; 2 Department of Criminology and Criminal Justice, Florida International University, PCA 261A, Miami, FL 33199 USA

**Keywords:** Mass shooting, Temperature anomaly, Routine activities theory, Temperature/aggression theories

## Abstract

Although researchers have found support for a relationship between temperature and violence and evidence of temporal patterns in violent crime, research on homicide shows less consistent results and no research on mass murder has been conducted. We address this by examining predictive factors in multi-victim shootings (those with four or more victims, including injured), a more general crime category than mass murder, but one with likely similar predictive factors. We used data from the Gun Violence Archive to understand the relationship between multi-victim shootings and temperature as well as other extrinsic factors. To avoid the confound between season and temperature, we employed temperature anomaly (the difference between actual and expected temperature) as a predictor of daily shooting rate. Using a generalized linear model for the daily count of multi-victim shootings in the U.S., we found that these events are significantly more frequent on weekends, some major holidays, hotter seasons, and when the temperature is higher than usual. Like other crimes, rates of multi-victim shooting vary systematically.

## Introduction

On October 1, 2017, the deadliest single-perpetrator mass shooting in U.S. history occurred in Las Vegas, leaving 58 victims dead and over 400 injured. These types of shocking events dominate public discourse on gun violence, garner significantly more media coverage than much more prevalent single-victim homicides, and are likely to impact public opinion disproportionately relative to public risk (Pinker, 2018). Many researchers (e.g., Slutkin, 2018) consider mass shootings to be a public health problem as well as a criminal justice issue. In the wake of events such as these, the focus tends to be on offender motivation. However, understanding broader, extrinsic patterns in the occurrence of violent crime may not only help researchers discern their causes, but also help criminal justice professionals prevent future crimes.

While there is considerable support for temperature effects, weekly cycles, and seasonal patterns in violent crime, research focusing on homicide shows less consistent results (e.g., Rotton & Cohn, 2002). Additionally, research has failed to distinguish between various forms of homicide, and there has been no research specifically examining how these factors may affect mass murder. In this study, we examine exogenous factors related to the incidence of multiple-victim shootings (MVSs), focusing specifically on temporal patterns and temperature variation.

## Temporal and Temperature Variations of Violent Crime

The scientific study of the relationship between temperature and aggression dates back to the nineteenth century, and involves three main types of research: randomized experiments, comparisons of rates of violence across geographic regions, and comparisons over time (Miles-Novelo & Anderson, 2019). All three types consistently show an increasing relationship between overall violent crime and temperature (see Rotton & Cohn, 2002, for an overview). Jacob, Lefgren, and Moretti (2006) reported that a 10 °F increase in average weekly temperature was associated with a 5% increase in violent crime, while Mares (2013) found that for each 1 °F above expected seasonal temperature, violence increased an average of 0.74%. Horrocks and Menclova (2011) found that violent crime increased with temperature until about 80 °F, after which point it began to decrease, while Gamble and Hess (2012) observed a temperature threshold of about 90 °F; these are similar to results obtained by Cohn and Rotton (2000) and Rotton and Cohn (2000). Heilmann and Kahn (2019) found violent crime in Los Angeles increased 5.7% on days with a maximum daily temperature greater than 85 °F, compared to days below that temperature. These relationships may be moderated by other factors, such as the time of day or the day of the week (e.g., Cohn & Rotton, 2005), as well as community income levels and access to climate control (e.g., Heilmann & Kahn, 2019; Rotton & Cohn, 2004). Research into seasonal patterns of violent crime also generally finds peaks during summer months, which have warmer temperatures (e.g., Breetzke & Cohn, 2012; McDowall, Loftin, & Pate, 2012). However, this relationship is confounded by seasonal behavioral changes.

The results of research examining the relationship between temperature and homicide is less consistent. McDowall et al. (2012) found intra-year crime variations of about 7% for homicide, but this was not statistically significant independent of season. Gamble and Hess (2012) found that a 1 °F increase in mean temperature was associated with less than a 0.1% increase in the homicide rate. Adding mean temperature to their baseline model explained only 0.1% of the variation in daily homicide rate. Maes, De Meyer, Thompson, Peeters, and Cosyns (1994) found no relationship between homicide and temperature in Belgium, and Yan (2000) found none in Hong Kong. The results of studies examining seasonal variations also lack consistency. While some reported increased homicide rates during summer months (e.g., Ceccato, 2005; McDowall et al., 2012), others have not found support for a seasonal pattern (e.g., Abel, Strasburger, & Zeidenberg, 1985; Yan, 2000). McDowall and Curtis (2015) also identified a December spike in homicides that nearly reached the July peak.

Weekends consistently see an increase in overall violent crimes (e.g., Harries, Stadler, & Zdorkowski, 1984; LeBeau & Corcoran, 1990), and homicide specifically (e.g., Abel et al., 1985; Ceccato, 2005). For example, Ceccato (2005) reported that over half the reported homicides in São Paulo occurred on weekends. Violent crimes are also more frequent on major holidays (Cohn & Rotton, 2003).

## Theoretical Background

The primary theories used to explain the link between temperature and violent behavior are Temperature/Aggression (T/A) theories and Routine Activities Theory (RAT). T/A theories (e.g., Anderson, Deuser, & DeNeve, 1995; Bell & Baron, 1976; Cohn, Rotton, Peterson, & Tarr, 2004) suggest that the relationship between aversive events and aggression is mediated by negative affect. Discomfort from higher temperatures is believed to increase irritability and frustration, leading to a greater likelihood of aggressive behavior, including crime; thus, seasonal changes in temperature explain seasonal crime patterns. T/A theories are supported by experimental laboratory research (Anderson et al., 1995; Bell & Baron, 1976) and field research showing reduced self-control at higher temperatures (see Gailliot, 2014, for a review).

RAT (Cohen & Felson, 1979) proposes a more indirect relationship between temperature and violence. It focuses on opportunities rather than motivation, emphasizing the ways in which temporal patterns structure behavior, particularly through changes in environmental conditions, many of which (e.g., temperature) are seasonal (e.g., Hipp, Bauer, Curran, & Bollen, 2004; McDowall et al., 2012). According to RAT, a crime requires three elements: a motivated offender, a suitable target, and the absence of a capable guardian against crime. RAT suggests that individuals have habitual behavior patterns (routine activities) that affect both criminal opportunity and victimization risk. Factors such as temperature, time of day, day of week, and holidays may affect discretionary routine activities (e.g., socializing), potentially affecting the likelihood that the three elements for crime will converge. Because warmer temperatures often encourage people to spend time outdoors, bringing them into closer proximity to others and increasing the potential for interaction between motivated offenders and suitable targets, RAT predicts that crime is likely to increase with temperature (Cohn & Rotton, 2000). RAT also supports a curvilinear relationship between temperature and crime; as temperatures become uncomfortably warm and create extreme discomfort, people may choose to spend more time indoors (e.g., in climate-controlled environments), reducing the possibility of interpersonal contact (Rotton & Cohn, 2000). Support for RAT over T/A theories is provided in research by Harp and Karnauskas (2018), which found higher rates of violent crime during mild compared to harsh winters. Mild winter temperatures increase the likelihood of outdoor activities, increasing the likelihood of interpersonal interactions and creating opportunities for crime.

## Our Study

Our goal was to identify factors associated with gun violence incidents involving multiple victims, rather than just those with multiple fatalities. Because the lethality of a violent encounter may be affected by contextual factors (e.g., Weaver et al., 2004), the shooter’s intent may be more accurately reflected by the number of victims shot rather than the number killed. The lethality perspective recommends that homicide research focus on “potentially lethal criminal actions rather than on completed homicides…” (Harris, Thomas, Fisher, & Hirsch, 2002, p. 156). We have adopted this perspective, focusing on multiple victim shootings (MVS) rather than only mass murders. We prefer the term “multiple victim shooting” to “mass shooting” since the latter is often confusingly used interchangeably with “mass murder.” We define an MVS to be a shooting event in which four or more victims (excluding the shooter) are killed or injured in a single location. The injured are included on an equal footing with fatalities because the factors influencing MVS event occurrence are unlikely to influence the number of fatalities versus injuries. Fox and Levin (2015, p. 12) support investigating such broad event scopes, stating that excluding incidents that do not reach preconceived murder counts “adds insult to injury for those victims.” Although mass murders differ in several ways from single-victim homicides, including showing greater evidence of premeditation (e.g., Fox & DeLateur, 2014; Fox & Levin, 1998), which may lead to different impacts of temperature and temporal factors on the two types of crimes, there has been no research focusing specifically on this issue.

We hypothesized that extrinsic factors known to influence violent crime rates—specifically weekends, holidays, cyclical variation in routine, and outside temperature—will also impact MVS rates. Quantifying these effects is complicated by the confounding of cyclical temporal factors and temperature: it is hotter outside in summer than winter. To help remedy this, we use temperature anomaly (the difference between actual temperature and that expected from seasonal variation) as a predictive factor in some models. This helps isolate the impact of weather-based temperature fluctuations, which we show to be non-multicollinear with temporal factors. Only a few studies of crime have examined temperature anomalies. Mares (2013) found a relationship between temperature anomaly and crime overall, but none for homicide. Williams, Hill, and Spicer (2015) found a positive relationship between temperature anomaly and assaults. Schinasi and Hamra (2017) found that violent crime was strongly associated with temperature deviations. Park et al. (2020) found that temperature anomaly was positively related to death by assault.

**Fig. 1:**
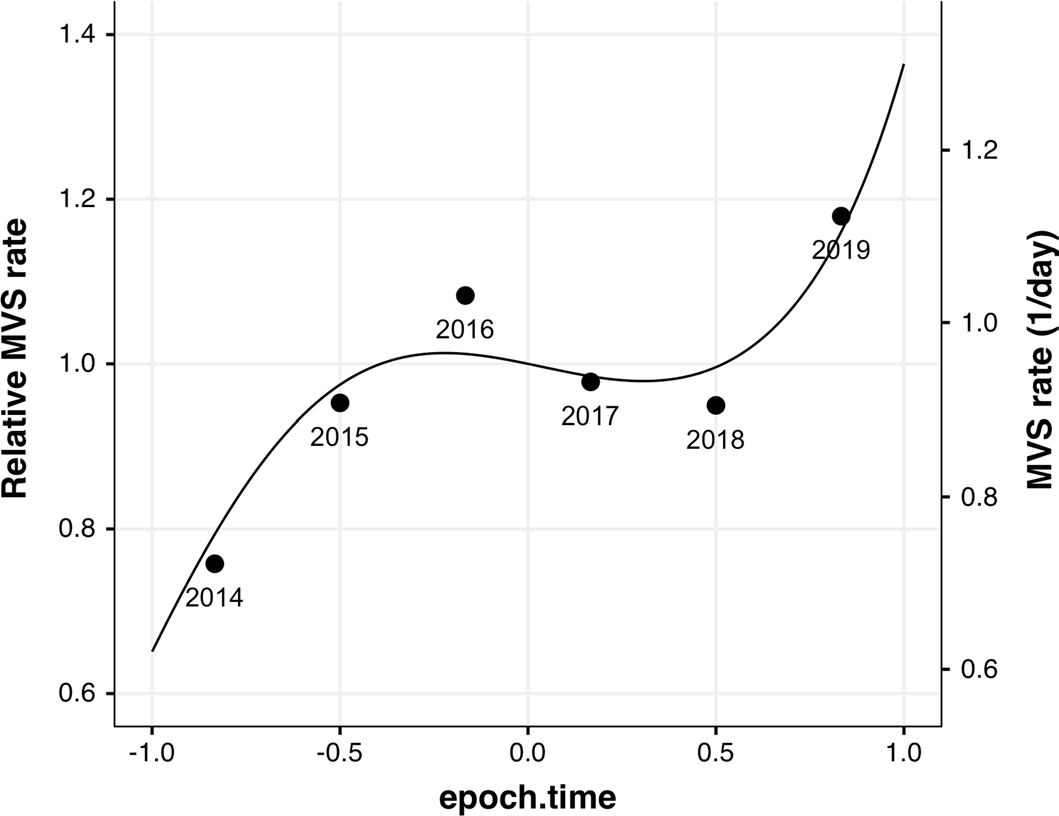
Multiple victim daily shooting rate averaged by year (points), with model M2 cubic fit to daily data (curve), demonstrating the data epoch’s daily MVS rate variation and its capture by the cubic fit. Relative rate (left scale) is referenced to *epoch.time* = 0

We obtained MVS data from the Gun Violence Archive^1^ (GVA), which collects information from law enforcement, government, and media sources. The GVA merges gang shootings, home invasion robberies, and familicide with traditional mass murder events. While studying each event type separately would be interesting and important, the dataset does not provide that information and it is beyond the scope of this broad exploratory research. We obtained data on MVSs in the continental U.S. from January 1, 2014 through December 31, 2019 (yielding a total 2191-day epoch). The data include the date, location, number killed, and number injured for each event. We aggregated events by day to produce a daily time-series of MVS rate (range = 0–7, mean = 0.95). From 2014 to 2019 the rate increased by more than half, from 0.7 to 1.1 per day (see Fig. 1). Whether this long-term change was due to an actual increase in MVS rate, crime reporting differences, or some other cause, we took it as given, and instead sought to understand how the day-to-day variation is influenced by extrinsic factors. We compared 12 models, all of which included effects for weekends and holidays, but differed in their accounting for outside temperature and yearly cyclical variation.

To model the effect of temperature on daily aggregated MVSs, we assessed a single aggregated daily temperature for the continental U.S. Although this spatially coarse measure does not necessarily reflect the conditions at any given MVS location, we show below that it has a strong relationship to MVS rate. Because temperature fluctuations tend to be correlated across much of the U.S. (data available upon request), using a summary value is reasonable as a first approximation. We obtained weather data from the National Centers for Environmental Information.^2^ For each weather station, we computed the hourly apparent temperature, accounting for both wind chill^3^ and temperature-humidity index (*heat.index* function in *weathermetrics R* package), and averaged these values in a weighted fashion by city to aggregate by day. Only temperatures^4^ for cities having at least one MVS in the dataset were included, weighted by the city’s total shooting count (see Fig. 3 in Appendix 2). In some models we employed the temperature anomaly, which is the difference between the day’s apparent temperature and its average for that week of the year (1–53). It has a mean of 0 and standard deviation (*SD*) of 5.5 °F that varies by month, from 2 °F in August to 8 °F in January (see Fig. 4 in Appendix 2). Due to missing temperature data, seven days^5^ were removed from the data set, leaving a total of 2184 days encompassing 2081 MVS events. In this pilot study, we do not include other weather factors (e.g., precipitation).

To account for cyclical variation in MVS rate, we optionally included a discrete factor of varying temporal granularity, be it season (four levels, with December the first month of winter), month (12 levels), or week (53 levels). Additionally, since there is a known relationship between major holidays and crime (e.g., Cohn & Rotton, 2003; Lester, 1987), we included a federal holiday factor (11 levels: New Year’s, Martin Luther King, Jr.’s Birthday, Washington’s Birthday, Memorial Day, Independence Day, Labor Day, Columbus Day, Veterans Day, Thanksgiving Day, Christmas, and none). For holidays typically celebrated across two days (New Year’s Eve and Day; Independence Day and early morning July 5th; Christmas Eve and Day), we assigned the holiday to both. We included a factor for the day being on a weekend. Finally, we accounted for year-to-year variation in MVS rate using a cubic polynomial in *epoch.time*, a continuous factor coded from − 1 to + 1 across the epoch 1/1/14 to 12/31/19.

## Results

To determine the effect of these extrinsic factors on daily MVS rate, we used a generalized linear model (GLM) with Poisson statistics. This model assumes that separate MVS events within a day are unrelated, and therefore only their expected rate can be predicted. Daily MVS counts are modeled as Poisson distributed at the day’s predicted rate. Because the event rate in the data is about one per day, statistical fluctuations in MVS count will be relatively large, making prediction of MVS count on any given day inaccurate. However, with sufficient data, we can determine which factors affect the expected MVS rate and quantify their impact.

We examined 12 models in a 4 × 3 factorial design that explored varying factors for annual cyclical effects (season, month, week, or none) and outside temperature (temperature, temperature anomaly, or none). We ranked models using the Akaike Information Criterion (AIC), which trades model accuracy for the number of model parameters (or degrees of freedom). In addition to possible cyclical temporal and temperature factors, each model included a cubic polynomial in *epoch.time* (we found no significant coefficients for higher-order terms at *p* < 0.05), a weekend factor, and a holiday factor. Results are shown in Table 1, with models (M1–M12) arranged from lowest to highest AIC^6^ (best to least supported model).

**Table 1:**
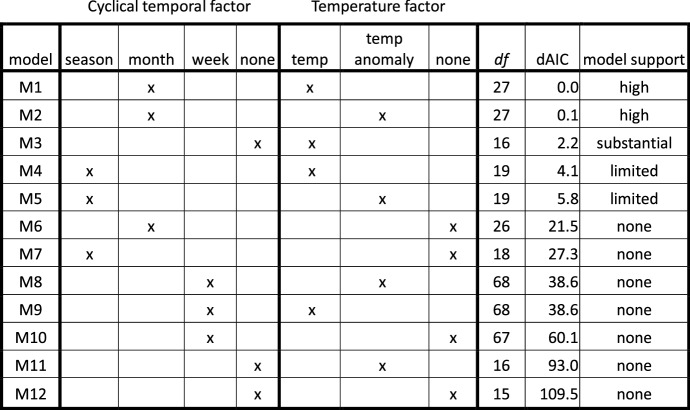
Performance comparison of models differing in cyclical temporal and temperature factors

*df* degrees of freedom, dAIC difference in Akaike information criterion statistic from lowest

The model rankings reveal some consistent findings. First, models without temperature factors (M6, M7, M10, and M12) all have dAIC values (differences from lowest AIC) of at least 21.6. A model with dAIC > 10 is considered highly unlikely to be the best (Burnham & Anderson, 2004), so models that include temperature are far superior to those that do not. M11 is also in this group, since it has knowledge of temperature anomaly, but no temporal reference to absolute temperature. Second, the top two models (M1 and M2) have an AIC difference of 0.1, which is considered insignificant. Both have month as the cyclical factor and take temperature into account, either absolutely (M1) or as its anomaly (M2). Since absolute temperature can be estimated knowing the month and the day’s temperature anomaly (as in M2), it is not surprising that these models perform equally well. Third, all models with week as the cyclical factor (M8–M10) have large dAICs, implying that including the additional parameters does not sufficiently improve prediction. Fourth, in contrast, season as the cyclical factor is too temporally granular (M4, M5). Finally, M3, which includes temperature but no temporal factor, has substantial support (dAIC = 2.2). This temperature model is much better than the month/no-temperature model M6 (dAIC = 19.3); including both factors gives a somewhat better model (M1).

**Fig. 2:**
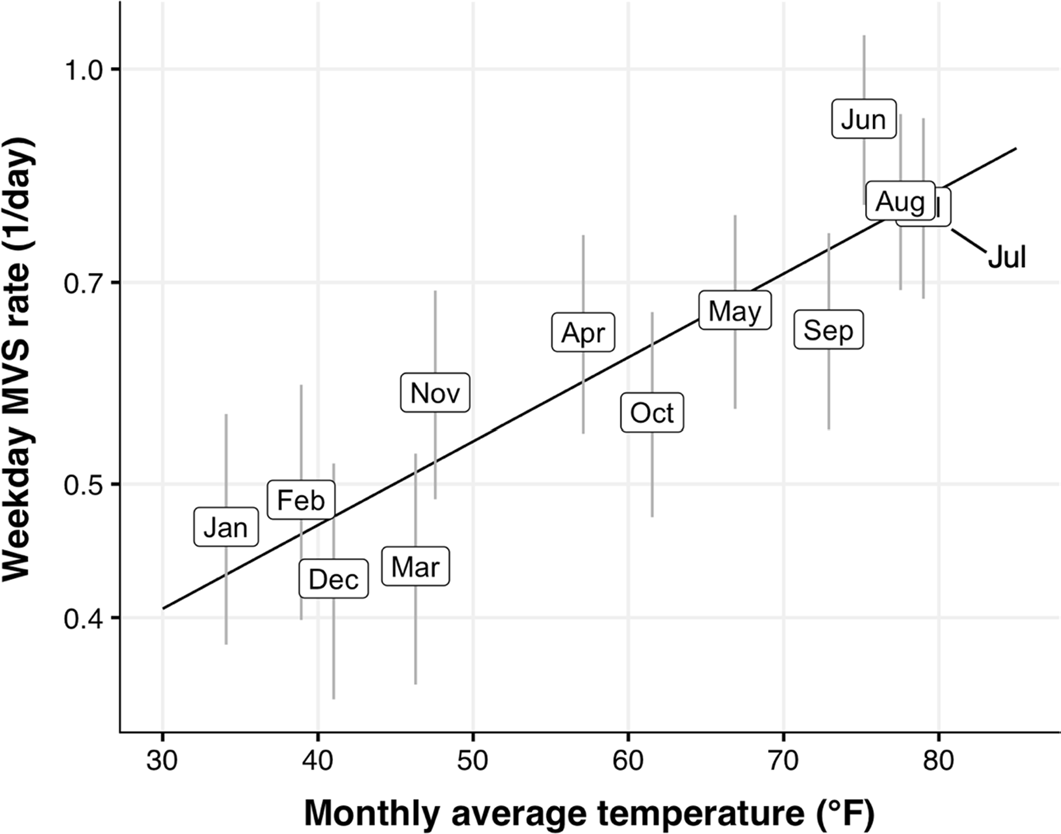
Model M2 exponentiated monthly coefficients (box centers) and 95% confidence intervals (vertical lines) plotted by average monthly apparent temperature, demonstrating significant association between monthly average temperature and corresponding weekday MVS rate model coefficients. Vertical axis is logarithmic; fit line is linear regression on semi-log scale

**Fig. 3:**
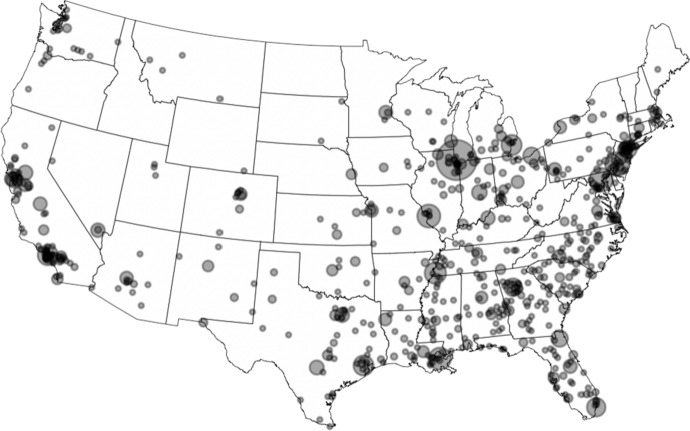
Geographic weightings of city apparent temperatures as combined to produce the continental U.S. apparent temperature. Circle areas are proportional to weighting. Circles are drawn partially transparent to reveal overlaps

To quantify these effects, we selected a high performing model with low multicollinearity for further investigation. This enables coefficients to be well estimated. Since M1 includes month and temperature factors, which are related and yield high variance inflation factor (VIF) (see Appendix 1), we chose M2, whose use of temperature anomaly reduces VIF (see Table 4 in Appendix 2). The full specification and diagnostics of M2 are found in Appendix 1. We did not find any significant two-factor interactions in this model (ANOVA, all *p*s greater than 0.05). Because the link function for Poisson GLMs is exponential, factors contribute multiplicatively to MVS rate. The *epoch.time* effect is shown in Fig. 1 (see Table 5 in Appendix 2 for coefficients); it approximates well the fluctuations in yearly average rate. The month factor coefficients are shown graphically in Fig. 2, exponentiated to quantify weekday MVS rate by month (mid-epoch) and plotted against average monthly temperature. Interestingly, the coefficients are well-approximated by a linear function of temperature (R^2^ = 0.79, *p* < 0.0001, with a slope of 0.014 ± 0.002 °F^−1^ [mean ± *SE*]; 95% CI 0.009, 0.019). Thus, hotter months have higher MVS rates (see Table 6 in Appendix 2 for coefficients).

Multiplicative effects of holidays and weekends are shown in Table 2. Three holidays have a significant effect (*p* < 0.05): Christmas, Independence Day, and New Year’s. Both Christmas and New Year’s (including their respective Eves) have MVS rates about twice as high as otherwise expected (95% CIs 1.2, 3.4 and 1.1, 3.2, respectively). Independence Day (including July 5th) has about three times the expected rate (95% CI 2.2, 4.4). Although all three of these significantly increased MVS holidays have two contributing days per year, their effects were significant even when modeled as single-day holidays. Finally, weekend days have 2.8 times the MVS rates of weekdays (95% CI 2.5, 3.0).

**Table 2:** Model M2

	*p* value	Multiplier	95% CI
Christmas Day	**0.01**	**2.0**	**1.18, 3.36**
Columbus Day	0.84	1.1	0.41, 2.99
Independence Day	**<** **0.0001**	**3.1**	**2.24, 4.38**
Labor Day	0.06	2.0	0.96, 3.97
Memorial Day	0.16	1.7	0.80, 3.66
MLK Jr.’s Birthday	0.12	2.0	0.83, 5.01
New Year’s Day	**0.02**	**1.9**	**1.12, 3.17**
Thanksgiving Day	0.81	1.1	0.42, 3.06
Veterans Day	0.69	1.2	0.52, 2.67
Washington’s Birthday	0.30	0.4	0.05, 2.53
Weekend	**<** **0.0001**	**2.8**	**2.54, 3.02**

Exponentiated coefficients (multipliers), their 95% confidence intervals, and *p* values for holidays and weekend two-level factors. Bolded values indicate statistical significance, *p* < 0.05

**Fig. 4:**
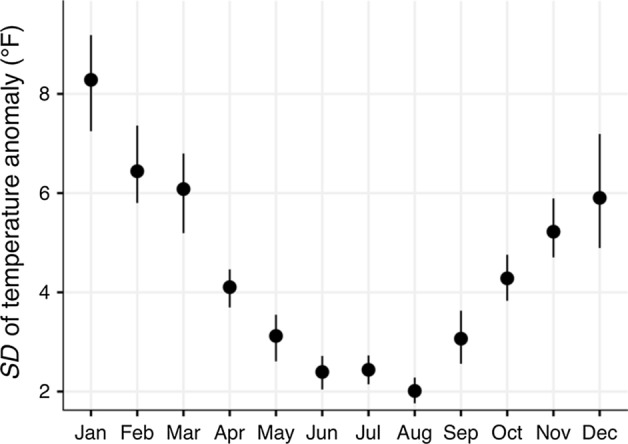
Intra-month standard deviation (*SD*) of temperature anomaly by month. Horizontal black line represents mean of intra-month *SD* across 6 years; bars represent bootstrap estimates of 95% confidence intervals from six data points (*mean_cl_boot* function from *R* package *Hmisc*)

Temperature anomaly (unexpectedly hot or cold days) alters the MVS rate exponentially by a rate of 0.022 ± 0.005 °F^−1^ (95% CI 0.013, 0.032), equivalent to 2.2% °F^−1^. Assuming a causal relationship, temperature anomaly fluctuations (± 1*SD* swing of 11 °F) would typically affect MVS by about 25%. Temperature anomaly deviates more in cold months than in warm ones (see Fig. 4 in Appendix 2), with estimated corresponding MVS fluctuations of minimally 10% in August and maximally 35% in January. By way of comparison, the M3 (temperature factor but no cyclical factor) absolute temperature coefficient is 0.015 ± 0.002 °F^−1^. This is not significantly different from the M2 temperature anomaly coefficient (*p* = 0.13, Gaussian test). Additionally, there is no significant difference within M2 between the linear slope relating monthly coefficient to temperature and the temperature anomaly coefficient (*p* = 0.10, Gaussian test). These temperature effects are somewhat larger than those reported by Jacob et al. (2006) and Mares (2013) for violent crime (0.006 and 0.007 °F^−1^, respectively), and of that reported by Park et al. (2020) for deaths by assault (0.006 °F^−1^).

## Discussion

This study extends the research into the effect of temporal and weather factors on crime, focusing on identifying extrinsic factors associated with daily MVS count in the continental U.S. It is the first study to examine the relationship between gun violence and temperature anomaly. Consistent with prior research into violent crime in general and homicide in particular, we found that the MVS rate is significantly related to the day of the week: MVS rates are nearly three times as frequent on weekend days than weekdays. Also consistent with prior violence research, some major holidays have higher MVS rates, specifically Christmas and New Year’s at double the rate, and Independence Day at triple. We examined whether the occurrence of televised major sporting events impacted the MVS rate, but found none for the Superbowl, World Series, or Olympics (data not shown).

The MVS rate exhibits cyclical temporal variation. It peaks in the summer months and is minimal in the winter ones, with a twofold total variation. Interestingly, the monthly model coefficients are well fit by an increasing linear relationship with temperature. Because of this month/temperature confound, we chose to use the temperature anomaly. We expect that a particular day being unexpectedly hot or cold to be independent of other factors (as borne out by M2’s VIF), so the temperature anomaly coefficient likely represents an isolated outside temperature effect. We found that the MVS rate is positively related to daily temperature anomaly, contributing a typical rate fluctuation of around 25%. Furthermore, the effect of temperature anomaly per °F is not significantly different either from that of temperature’s impact on the month coefficient or from the absolute temperature coefficient in a model that excludes cyclical temporal variation (M1). Taken together, these effects would render an unusually hot weekend Independence Day an order of magnitude riskier than a typical summer weekday.

The positive relationship between temperature anomaly and MVS is consistent with expectations from both T/A Theory and RAT. Additionally, the effects of cyclical temporal factors on MVS are consistent with predictions derived from RAT. MVS events are significantly more likely to occur on weekends and some major holidays, times when routine activities show greater variability.

One limitation of this study is its use of a single weighted temperature anomaly rather than regional values. The available data are not sufficiently large to power such regional investigation. This study was also limited by the need to obtain MVS data from an independent agency, rather than from official crime statistics. Finally, the information included in the dataset was limited. It did not include the time of each shooting, making it difficult to identify shootings that occurred just after midnight and might be related to the previous day; nor was there information on the victim/offender relationship, making it impossible to separate domestic and non-domestic shootings.

While gun violence is generally viewed as a criminal justice issue, it is a leading cause of injury and death in the U.S. MVSs may account for a small percentage of gun-related deaths, but their effect on society is far-reaching. Expanding our understanding of MVS, including greater awareness of the relationship between MVS and factors such as temperature, may help to provide law enforcement with the tools needed to anticipate MVS events and prevent some of the resulting injuries and deaths.

## Figures and Tables

**Fig. 5:**
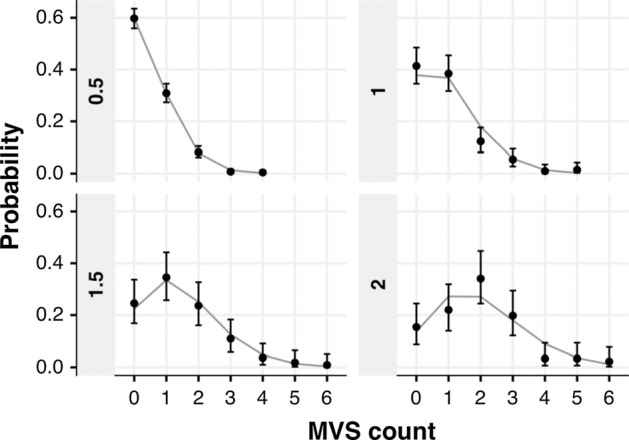
Conditional distributions of MVS count at four model-predicted MVS rates. Data are pooled within each graph for predicted rates within 0.25 of the stated rate (0.5, 1, 1.5, or 2 MVS events per day). Points represent relative frequencies of MVS counts, and bars represent 95% confidence intervals (binomial distribution). Lines correspond to Poisson probability distributions of the graph’s stated rate

## Appendix 1: Statistical Methods

Analyses were performed using *R*^7^ 3.6*.1* on *macOS* 10.14.6. The function *glm* was used to model the daily number of MVS events as Poisson distributed (log link function), based on a linear model. Model M2 included a continuous predictor for *temp.anomaly,* a cubic polynomial in *epoch.time*, and discrete factors for *Weekend* (True/False), *Holiday* (11 levels; see text), and *Month* (12 levels). This model was specified as:

$$ {\text{glm}}\left( {{\text{MVS}}.{\text{count}}\,\sim \,\left( {{\text{Month}} - 1} \right) + {\text{poly}}\left( {{\text{epoch}}.{\text{time}},3,{\text{raw}} = {\text{TRUE}}} \right) + {\text{Holiday}} + {\text{Weekend}} + {\text{temp}}.{\text{anomaly}},{\text{ family}} = {\text{poisson}}} \right) $$

We evaluated a number of diagnostics of model applicability. To ensure the model did not exhibit multicollinearity, we employed the *vif* function on the model adjusted to contain an intercept, and examined the GVIF^1/(2**df*)^ values for each factor. All were 1.03 or less (see Table 4 in Appendix 2), indicating absence of multicollinearity. In contrast, M1 had a GVIF^1/(2**df*)^ value of 3 for the temperature factor, indicating multicollinearity (with month). We visually compared the conditional distributions of MVS count to the expected Poisson distributions, and found them to fit well (see Fig. 4 in Appendix 2), validating the Poisson assumption. We further checked for zero inflated data, which we did not observe (model *p*(0) = 0.44, data *p*(0) = 0.45). We tested for over-dispersion using the *dispersiontest* function, which failed to reject the null hypothesis of unit dispersion (*p* = 0.20). To validate the assumption of temporally uncorrelated shooting rates across days, we tested for correlated model residuals using the Ljung–Box method (*Box.test* function), which found no significant correlation (*p* = 0.71).

Model parameter estimates, standard errors, and *p* values were extracted using the *summary.glm* function. Their 95% CIs were computed using the *confint.lm* function.

Packages and functions used are listed in Table 3.

**Table 3:** R packages, versions, functions, and data used in this study

Package	Version	*functions*/data
AER	1.2.8	*dispersiontest*
binom	1.1.1	*binom.confint*
car	3.0.6	*outlierTest, Anova*
caTools	1.17.1.3	*runmean*
Hmisc	4.3.0	*mean_cl_boot*
noncensus	0.1	Zip codes
RANN	2.6.1	*nn2*
timeDate	3043.102	Holiday dates
weathermetrics	1.2.2	*heat.index*

## Appendix 2

See Figs. 3, 4 and 5 and Tables 4, 5 and 6.

**Table 4:** Variance inflation factor results for the model M2

	GVIF	*df*	GVIF^1/(2**df*)^
Month	1.89	11	1.03
Poly(epoch.time, 3, raw = TRUE)	1.13	3	1.02
Holiday	1.75	10	1.03
Weekend	1.02	1	1.01
Temp.anomaly	1.07	1	1.03

*GVIF* generalized variance inflation factor, *df* degrees of freedom

**Table 5:** Coefficients of polynomial fit to log of MVS rate across the epoch 1/1/14 to 12/31/19, with *epoch.time* correspondingly ranging from − 1 to + 1

	*p* value	Coefficient	95% CI
epoch.time	0.336	− 0.09	− 0.29, 0.10
(epoch.time)^2^	0.445	− 0.06	− 0.21, 0.09
(epoch.time)^3^	0.003	0.46	0.16, 0.77

**Table 6:** Estimated weekday MVS rate and their 95% confidence intervals by month from model M2, corresponding to the time point *epoch.time* = 0

Month	Weekday rate (1/day)	95% CI
Jan	0.5	0.38, 0.56
Feb	0.5	0.40, 0.59
Mar	0.4	0.36, 0.53
Apr	0.6	0.55, 0.76
May	0.7	0.57, 0.79
Jun	0.9	0.80, 1.06
Jul	0.8	0.68, 0.92
Aug	0.8	0.69, 0.93
Sep	0.6	0.55, 0.76
Oct	0.6	0.47, 0.67
Nov	0.6	0.49, 0.69
Dec	0.4	0.35, 0.52

## Footnotes

1 https://www.gunviolencearchive.org/reports, Downloaded 1/3/2020.

2 https://www.ncei.noaa.gov/data/local-climatological-data/, downloaded 11/19/2019 (2014–2018 data) and 1/4/20 (2019 data).

3 https://en.wikipedia.org/wiki/Wind_chill; we use the formula T_WC_ = 35.74 + 0.6215 * T_DB_ − 35.75 * v^0.16^ + 0.4275 * T_DB_ * v^0.16^, with T_DB_ the dry bulb temperature and v the wind speed in MPH. All temperatures are in °F. Wind chill correction was performed for T_DB_ < 50 °F and v > 3 MPH.

4 City apparent temperatures were estimated as averages of the up to nearest six weather stations within 250 miles of the city’s location (defined by the location of the lowest numerical zip code assigned to the city, found through the *R* package *concensus*). Daily apparent temperature for each station were evaluated on an hourly basis and averaged across 24 h in a day, accepting at most one data point as not available.

5 4/14/15, 11/19/16, 12/18/16, 2/9/17, 2/5/18, 4/8/18, and 2/16/19.

6 We also evaluated the Bayesian Information Criterion (BIC), which greatly penalizes additional parameters when modeling large data sets, such as this one. BIC very strongly selected the model M3, with a temperature factor but no temporal cyclical factor, above all others. We believe BIC to be too conservative with model parameters, and prefer AIC for model ranking.

## References

[CR1] Abel EL, Strasburger EL, Zeidenberg P (1985). Seasonal, monthly, and day-of-week trends in homicide as affected by alcohol and race. Alcoholism: Clinical and Experimental Research.

[CR2] Anderson CA, Deuser WE, DeNeve KM (1995). Hot temperatures, hostile affect, hostile cognition, and arousal: Tests of a general model of affective aggression. Personality and Social Psychology Bulletin.

[CR3] Bell PA, Baron RA (1976). Aggression and heat: The mediating role of negative affect. Journal of Applied Social Psychology.

[CR4] Breetzke GD, Cohn EG (2012). Seasonal assault and neighborhood deprivation in South Africa: Some preliminary findings. Environment and Behavior.

[CR5] Burnham KP, Anderson DR (2004). Multimodel inference: Understanding AID and BIC in model selection. Sociological Methods and Research.

[CR6] Ceccato V (2005). Homicide in São Paulo, Brazil: Assessing spatial-temporal and weather variations. Journal of Environmental Psychology.

[CR7] Cohen LE, Felson M (1979). Social change and crime rate trends: A routine activity approach. American Sociological Review.

[CR8] Cohn EG, Rotton J (2000). Weather, seasonal trends and property crimes in Minneapolis, 1987–1988: A moderator-variable time-series analysis of routine activities. Journal of Environmental Psychology.

[CR9] Cohn EG, Rotton J (2003). Even criminals take a holiday: Instrumental and expressive crimes on major and minor holidays. Journal of Criminal Justice.

[CR10] Cohn EG, Rotton J (2005). The curve is still out there: A reply to Bushman, Wang, and Anderson’s (2005) “Is the curve relating temperature to aggression linear or curvilinear?”. Journal of Personality and Social Psychology.

[CR11] Cohn EG, Rotton J, Peterson AG, Tarr DB (2004). Temperature, city size, and the Southern subculture of violence: Support for social escape/avoidance (SEA) theory. Journal of Applied Social Psychology.

[CR12] Fox JA, DeLateur MJ (2014). Mass shootings in America: Moving beyond Newtown. Homicide Studies.

[CR13] Fox JA, Levin J, Tonry M (1998). Multiple homicide: Patterns of serial and mass murder. Crime and justice.

[CR14] Fox JA, Levin J (2015). Mass confusion concerning mass murder. The Criminologist.

[CR15] Gailliot MT (2014). An assessment of the relationship between self-control and ambient temperature: A reasonable conclusion is that both heat and cold reduce self-control. International Review of Social Sciences and Humanities.

[CR16] Gamble JL, Hess JJ (2012). Temperature and violent crime in Dallas, Texas: Relationships and implications of climate change. Western Journal of Emergency Medicine.

[CR17] Harp RD, Karnauskas KB (2018). The influence of interannual climate variability on regional violent crime rates in the United States. GeoHealth.

[CR18] Harries KD, Stadler SJ, Zdorkowski RT (1984). Seasonality and assault: Explorations in inter-neighborhood variation, Dallas 1980. Annals of the Association of American Geographers.

[CR19] Harris AR, Thomas SH, Fisher GA, Hirsch DJ (2002). Murder and medicine: The lethality of criminal assault 1960-1999. Homicide Studies.

[CR20] Heilmann, K., & Kahn, M. E. (2019). *The urban crime and heat gradient in high and low poverty areas*. NBER working paper no. 25961. National Bureau of Economic Research, Cambridge, MA.

[CR21] Hipp JR, Bauer DJ, Curran PJ, Bollen KA (2004). Crimes of opportunity or crimes of emotion? Testing two explanations of seasonal change in crime. Social Forces.

[CR22] Horrocks J, Menclova AK (2011). The effects of weather on crime. New Zealand Economic Papers.

[CR23] Jacob B, Lefgren L, Moretti E (2006). The dynamics of criminal behavior: Evidence from weather shocks. The Journal of Human Resources.

[CR24] LeBeau JL, Corcoran WT (1990). Changes in calls for police service with changes in routine activities and the arrival and passage of weather fronts. Journal of Quantitative Criminology.

[CR25] Lester D (1987). Suicide and homicide on national holidays. Psychological Reports.

[CR26] Maes M, De Meyer F, Thompson P, Peeters D, Cosyns P (1994). Synchronized annual rhythms in violent suicide rate, ambient temperature and the light-dark span. Acta Psychiatrica Scandinavica.

[CR27] Mares D (2013). Climate change and levels of violence in socially disadvantaged neighborhood groups. Journal of Urban Health: Bulletin of the New York Academy of Medicine.

[CR28] McDowall D, Curtis KM (2015). Seasonal variation in homicide and assault across large U.S. cities. Homicide Studies.

[CR29] McDowall D, Loftin C, Pate M (2012). Seasonal cycles in crime, and their variability. Journal of Quantitative Criminology.

[CR30] Miles-Novelo A, Anderson CA (2019). Climate change and psychology: Effects of rapid global warming on violence and aggression. Current Climate Change Reports.

[CR31] Park RM, Bennett JE, Tamura-Wicks H, Kontis V, Toumi R, Danaei G, Ezzati M (2020). Anomalously warm temperatures are associated with increased injury deaths. Nature Medicine.

[CR32] Pinker, S. (2018, February 17). *The media exaggerates negative news. This distortion has consequences*. The Guardian. Retrieved December 6, 2018, from https://www.theguardian.com/commentisfree/2018/feb/17/steven-pinker-media-negative-news.

[CR33] Rotton J, Cohn EG (2000). Violence is a curvilinear function of temperature in Dallas: A replication. Journal of Personality and Social Psychology.

[CR34] Rotton J, Cohn EG, Bechtel RB, Churchman A (2002). Climate, weather and crime. Handbook of environmental psychology.

[CR35] Rotton J, Cohn EG (2004). Outdoor temperature, climate control, and criminal assault: The spatial and temporal ecology of violence. Environment and Behavior.

[CR36] Schinasi LH, Hamra GB (2017). A time series analysis of associations between daily temperature and crime events in Philadelphia, Pennsylvania. Journal of Urban Health.

[CR37] Slutkin, G. (2018, February 21). *Let’s treat these mass shootings like the public health crisis that they are*. Retrieved December 30, 2018, from https://thehill.com/opinion/criminal-justice/374719-lets-treat-these-mass-shootings-like-the-public-health-crisis-that.

[CR38] Weaver GS, Wittekind JEC, Huff-Corzine L, Corzine J, Petee TA, Jarvis JP (2004). Violent encounters: A criminal event analysis of lethal and nonlethal outcomes. Journal of Comparative Criminal Justice.

[CR39] Williams MN, Hill SR, Spicer J (2015). The relationship between temperature and assault in New Zealand. Climatic Change.

[CR40] Yan YY (2000). Weather and homicide in Hong Kong. Perceptual and Motor Skills.

